# Editorial for Special Issue “Current Advances in Oxytocin Research”

**DOI:** 10.3390/cimb47050363

**Published:** 2025-05-15

**Authors:** Claudia Camerino

**Affiliations:** 1 Department of Precision and Regenerative Medicine, School of Medicine, University of Bari Aldo Moro, P.za G. Cesare 11, 70100 Bari, Italy; ccamerino@libero.it; 2 Department of Physiology and Pharmacology “V. Erspamer”, Sapienza University of Rome, P.le Aldo Moro 5, 00185 Rome, Italy

Very few hormones and neurotransmitters are as fascinating as oxytocin. The discovery of oxytocin dates back to 1955, when Professor Vincent du Vigneaud was awarded the Nobel Prize in Chemistry for sequencing oxytocin [1]. This achievement marked the culmination of a long line of research initiated in 1895. For the following 50 years or so, oxytocin was primarily studied for its role in uterine contractility and milk ejection during lactation. However, in the early 2000s, its physiological functions were reconsidered. Researchers discovered that oxytocin also plays a role in glucose metabolism and obesity. Notably, a lack of oxytocin was found to contribute to the onset of obesity and metabolic syndrome, even with no changes in food intake [2,3]. Since then, thousands of studies have explored the wide range of beneficial physiological effects associated with oxytocin. The oxytocin receptor, a G-protein coupled receptor, has been shown to exhibit sexual dimorphisms, indicating that its role is not exclusive to the female gender and, by extension, not limited to uterine contraction. But what is the real physiological identity of oxytocin? The answer begins with the early evidence showing that oxytocin is involved in thermoregulation [4,5,6] and muscle contraction [7,8]. Indeed, like many hypothalamic hormones, oxytocin regulates thermogenesis, food intake, and reproduction—three fundamental processes essential to life. Oxytocin plays a distinctive role in regulating muscle contraction and thermoregulation. This collection of articles was conceived to provide readers with a comprehensive overview of the physiological functions of oxytocin in both health and disease. A total of eight articles are featured in this Special Issue, including two original research articles that explore the novel physiological functions of oxytocin. One study found that the hindbrain administration of oxytocin reduced body weight and energy intake, while stimulating the temperature of interscapular brown adipose tissue in male mice, in which obesity had been induced through diet. In contrast, in female rats fed a high-fat diet, the increase in thermogenesis and weight loss elicited by oxytocin occurred independently of the sympathetic innervation to brown adipose tissue. Moreover, in an in vitro model of oxygen-glucose deprivation, oxytocin was found to confer a degree of neuroprotection, specifically in cases of severe lesions in hippocampal neurons after 7 days in vitro. This findings offer new therapeutic possibilities for the prevention of perinatal asphyxia and hypoxic-ischemic encephalopathy. The remaining six articles in this Special Issue are reviews. Three of them discuss the role of oxytocin in Prader–Willi syndrome and autism spectrum disorder, highlighting the importance of oxytocin’s pharmacological properties for the treatment of several diseases, including obesity, Prader–Willi syndrome, and autism spectrum disorder [9,10,11]. The fourth review article examines the role of oxytocin in mesodermal stem cell-derived lineages. The fifth review investigates the presence of possible alterations in oxytocin levels in polycystic ovary syndrome, noting that serum oxytocin levels are reported to be lower in affected individuals. Finally, the sixth review article introduces the innovative concept of hormesis and the role of oxytocin and vasopressin in the physiological adaptation to stressor events. In conclusion, it was most gratifying to receive such enthusiastic responses from all the contributors, who submitted excellent work in both basic and translational research, as well as from the reviewers and the editorial team. The completion of this collection paves the way for new opportunities and collaborations. It was an honour to serve as Guest Editor, and I extend my sincere congratulations to all the contributors for finalizing this collection of articles, published in commemoration of 70th anniversary of oxytocin’s discovery (1955–2025).
